# Neural, Cellular and Molecular Mechanisms of Active Forgetting

**DOI:** 10.3389/fnsys.2018.00003

**Published:** 2018-02-06

**Authors:** Jorge H. Medina

**Affiliations:** ^1^Laboratorio de Memoria, IBCN Universidad de Buenos Aires-Consejo Nacional de Investigaciones Científicas y Técnicas (UBA-CONICET), Buenos Aires, Argentina; ^2^Departamento de Fisiología, Facultad de Medicina, Universidad de Buenos Aires, Buenos Aires, Argentina

**Keywords:** memory, forgetting, *Drosophila*, dopamine, rac 1, hippocampus, BDNF, AMPA receptors

## Abstract

The neurobiology of memory formation attracts much attention in the last five decades. Conversely, the rules that govern and the mechanisms underlying forgetting are less understood. In addition to retroactive interference, retrieval-induced forgetting and passive decay of time, it has been recently demonstrated that the nervous system has a diversity of active and inherent processes involved in forgetting. In *Drosophila*, some operate mainly at an early stage of memory formation and involves dopamine (DA) neurons, specific postsynaptic DA receptor subtypes, Rac1 activation and induces rapid active forgetting. In mammals, others regulate forgetting and persistence of seemingly consolidated memories and implicate the activity of DA receptor subtypes and AMPA receptors in the hippocampus (HP) and related structures to activate parallel signaling pathways controlling active time-dependent forgetting. Most of them may involve plastic changes in synaptic and extrasynaptic receptors including specific removal of GluA2 AMPA receptors. Forgetting at longer timescales might also include changes in adult neurogenesis in the dentate gyrus (DG) of the HP. Therefore, based on relevance or value considerations neuronal circuits may regulate in a time-dependent manner what is formed, stored, and maintained and what is forgotten.

## Introduction

In general sense forgetting refers to as the inability to express some information acquired previously, no matter whether or not that information establishes a retrievable memory. In strict sense forgetting is referred to as the inability to recall something now that could be retrieved on an earlier occasion. This could happen if an established memory is no longer available. In other words, that memory is lost. In addition, the possibility exists that forgetting is due to a temporary failure in accessing to that memory: in other words, a deficit in memory retrieval. However, forgetting must be differentiated from amnesia. Both have memory loss but while forgetting is a natural process, amnesia is a pathological one.

Two main ideas about forgetting emerged in the field of experimental psychology. One dominant hypothesis postulates that interference at different steps of memory processing is the principal cause of forgetting (Wixted, 2004). This interference could be at encoding, or at the formation and consolidation or even at the moment of retrieving a memory (Skaggs, 1933). For instance, Wixted (2004) suggests that the amnesic effect of a new learning on previously encoded material (retroactive interference) is mainly due to the use of the resources available to consolidate the original trace. The immediate early gene Arc is one of the resources involved in memory competition (Martínez et al., 2012). The second hypothesis postulates that passive or active decay of the memory trace is also responsible to forgetting. This idea has been consistently criticized during many years (Neath and Brown, 2012). However, some recent neurobiological data regarding cellular and molecular mechanisms of long-term memory (LTM) persistence and forgetting appears to endorse the idea that active forgetting is an important part in determining the fate of memories (see below). A detailed description of biological and non-biological arguments supporting the idea that the brain is endowed with intrinsic forgetting processes is provided by two recent review articles (Hardt et al., 2013; Davis and Zhong, 2017).

During the last five decades a great body of evidence accumulated regarding the neurobiology of memory formation, and its main consequences extinction and reconsolidation (McGaugh, 2000; Sara, 2000; Kandel, 2001; Dudai, 2002; Morris, 2006). However, much less attention has been focused on the mechanisms of active forgetting (Davis and Zhong, 2017). Without forgetting you may have in mind all the information from the external world, including those data which are not relevant. As the argentinian writer Jorge Luis Borges pointed out in his story “Funes el memorioso” (Funes the memorizer), if one acquires and stores all the information we experience in a given day, we will take almost another day to retrieve it, and therefore we will learn nothing at all in such a day. The sentences “Memories are islands in an ocean of forgetting” and “Memory: name we give to the cracks of stubborn forgetting” are two wonderful, poetic but realistic views of what Borges and many neuroscientists in the last 50 years consider memory and forgetting: forgetting of learned experiences is, at least, as important as the process of making memories.

Although there are several types of forgetting including retroactive interference (Wixted, 2004), retrieval-induced forgetting (Anderson, 2003), passive decay of memory and active or intrinsic forgetting (see the following reviews for more details and references, Frankland et al., 2013; Hardt et al., 2013; Davis and Zhong, 2017), in this review I will focus on what is known as active forgetting. In a general sense active forgetting is the inherent neural, cellular and molecular processes involved in erasing the substrate of a memory or in suppressing its accessibility. From a historical point of view I will first present some indirect evidence for the existence of a natural and active process of forgetting. This experimental evidence is however inconclusive and fragmentary. Therefore, the main focus of this article will be on what it is considered the direct evidence of active inherent forgetting, describing mechanisms of active forgetting in *Drosophila* and in rodents. Direct evidence is referred to the demonstration of a mechanism or a sequence of events that is required for and sufficient to erase the substrate of a given memory. Finally, some of the processes that modulate the rate of active forgetting will be briefly described.

## Indirect Evidence for Active Forgetting

During the last 15 years a wealth of indirect evidence supports the idea that an inherent active mechanism of forgetting is present in the central nervous system. Studies analyzing the cellular and molecular underpinnings responsible for establishing remote memories and for their persistence shed new light for a better understanding of active forgetting process of consolidated memories (see for references Katche et al., 2013a,b; Bekinschtein et al., 2014; Katche and Medina, 2017). Forgetting of very short-lived memories including the so-called immediate memory (McGaugh, 2000), and also forgetting due to suppression of memory expression are not considered in this review; in addition, in order to avoid confounds between promotion of forgetting with inhibition of memory consolidation or inhibition of forgetting with facilitation of memory consolidation, all the findings described in this section mainly involve specific modifications in the durability of consolidated LTM without modifications in acquisition, memory formation, or retrieval (see also below “predictions and requirements” in direct evidence):

1. Active forgetting of long-lasting contextual fear memory was achieved by late posttraining temporary knocking down of NMDA receptors or alpha-CaMKII activity in the forebrain (Wang et al., 2003; Cui et al., 2004). These findings are consistent with those showing that CaMKII heterozygous knockout mice exhibited rapid forgetting of remote, but not recent LTM (Frankland et al., 2001). Recently, chronic inhibition of the NMDA receptor and L-type voltage-dependent Ca^2+^ channel maintains long-term object location memory that otherwise would have been forgotten (Sachser et al., 2016).
2. Selective forgetting of remote memories was found after manipulation of several protein kinases. For instance, ERK1/2 participates late after training to sustain LTM storage of two different hippocampus (HP)-dependent learning tasks (Bekinschtein et al., 2008; Eckel-Mahan et al., 2008). A circadian oscillation of the phosphorylation state of ERK1/2 in the HP has been implicated in memory persistence (Eckel-Mahan, 2012). Inducible and targeted deletion of hippocampal ERK5 induced a specific impairment in remote avoidance memory (Pan et al., 2012). Shan et al. (2008) demonstrated that knocking down adenylyl cyclases 1 impaired remote contextual fear memory. Adenylyl cyclases 1 and 8 regulate long-lasting transcriptional changes in the HP important for memory persistence (Wieczorek et al., 2010). Most of these genes are up-regulated in wild-type mice 48 h after training. These authors found that knocking-down adenylyl cyclase 8 provoked active forgetting of remote memory. Finally, selective forgetting of well-consolidated memories was observed after blockade of PKMζ (Sacktor, 2011). PKMζ appears to keep memory storage by regulating GluA2-dependent AMPA receptor trafficking (Migues et al., 2010). This atypical PKC isoform is persistently activated during L-LTP and was repeatedly suggested to be involved in maintenance of memory storage long after memory is consolidated into LTM (Sacktor, 2011). It has been shown that PKMζ maintains object recognition memory for about 1 week preventing the internalization of GluA2-containing AMPA receptors in the dorsal HP (Migues et al., 2010). The inhibition of synaptic removal of these hippocampal receptors supports memory persistence of consolidated object location and food-reward conditioned place preference without altering acquisition or memory formation (Migues et al., 2016). Therefore, it was proposed that endocytosis of GluA2-containing AMPA receptor in activated synapses appears to be one mechanisms of time-dependent forgetting in learning tasks with different valence. However, it is important to mention here that to fully accomplish predictions about an active forgetting mechanism it remains to be determined whether the facilitation of synaptic removal of GluA2-containing AMPA receptors induces forgetting of consolidated memories.
3. Rapid forgetting without impairments in memory formation or retrieval of long-lasting aversive/fear memories was consistently observed after inhibition of protein synthesis in the dorsal HP, amygdala, medial prefrontal, retrosplenial or insular cortices late after training (Bekinschtein et al., 2007; Ou et al., 2010; Martínez-Moreno et al., 2011; Katche et al., 2012; Gonzalez et al., 2013; Tomaiuolo et al., 2014). Twelve hours after training, BDNF was required and sufficient to sustain LTM storage for many days (Bekinschtein et al., 2007, 2008). In other words, BDNF attenuates forgetting. BDNF, via activation of TrKB receptors, ERK1/2 phosphorylation and c-fos, arc, and zif-268 expression promoted the establishment of a long-lasting fear LTM (Sweatt, 2001; Bekinschtein et al., 2008; Katche et al., 2010; Tomaiuolo et al., 2014; Nakayama et al., 2015, 2016). Inhibition of any of the molecular steps of this signaling cascade late after training induced rapid forgetting of LTM. More recently, the group of Alberini (Taubenfeld et al., 2001a; Bambah-Mukku et al., 2014) demonstrated that protein synthesis and BDNF signaling are required for at least 24 h after training to maintain avoidance memory storage in rats. Several afferent systems to the ventral tegmental area including those coming from the lateral habenula, the medial prefrontal cortex and the pedunculopontine tegmental nucleus may regulate temporal stability of consolidated fear LTM through the modulation of dopamine (DA)/BDNF signaling pathway in the HP (Lima et al., 2013; Gonzalez et al., 2014; Tomaiuolo et al., 2014).
  What are the upstream neurotransmitters that trigger or modulate BDNF signaling to maintain long-lasting memories? DA in the dorsal HP has a critical role in the duration of LTM storage (Rossato et al., 2009). Long-lived fear memory vanished rapidly when the D1-DA receptor antagonist SCH23390 was injected into the dorsal HP 12 h after inhibitory avoidance training. On the other hand, delivery of the D1 agonist SK38393 at the same critical posttraining time converted a rapidly decaying fear LTM in a persistent one. NMDAr activation in the VTA upregulates the hippocampal dopaminergic system which through a D1-dependent mechanism controls the expression of BDNF required for LTM persistence (Rossato et al., 2009). Other extracellular signals that modulate the durability of memories via regulation of that late consolidation phase are noradrenaline (Katche et al., 2010; Mello-Carpes et al., 2016), serotonin (Slipczuk et al., 2013), glutamate (Rossato et al., 2009), acetylcholine (Parfitt et al., 2012; Porto et al., 2015), insulin-like growth factor 2 (Lee et al., 2015), spermidine (Signor et al., 2014), and the addictive drug nicotine (Lima et al., 2013).
4. Several genes or mechanisms that regulated transcription and translation have been also showed to selectively modify the duration of LTM without alterations in other stages of memory processing (Taubenfeld et al., 2001b. It has been reported that integrin beta 2 and Sterol O-acyltransferase 1 are two genes required for long-lasting contextual fear LTM tested 7 days after training, but not for short-lasting fear LTM tested 1 day posttraining (Matynia et al., 2008). Cytoplasmic polyadenylation element binding protein (CPEB), a molecule that activates dormant mRNAs, is required for the persistence but not formation of long-term facilitation (LTF) in *Aplysia*. A late phase of sustained CPEB prion-like multimer activity is also needed for the synaptic growth associated with persistent LTF in a local protein synthesis-dependent manner (Miniaci et al., 2008; Si et al., 2010). Genetic ablation of CPEB3 in mice induced active forgetting by impairing the maintenance of both hippocampal long-term potentiation and HP-dependent spatial memory (Fioriti et al., 2015).

Epigenetic mechanisms also contribute to modulate memory persistence and forgetting. It has been suggested that a shift in the regulatory balance activating NFkB transcription factor and histone acetylation is sufficient to render a memory more persistent (Federman et al., 2013). Briefly, inhibition of hippocampal histone acetyltransferases during consolidation of an object recognition task induced rapid forgetting while inhibition of hippocampal histone deacetylases, that normally silence transcription, induced persistent recognition memory (Stefanco et al., 2009; Federman et al., 2013). It has been also demonstrated that DNA methylation in the medial prefrontal cortex plays a critical role in the durability of fear memory (Miller et al., 2010). The authors found that a single contextual fear learning experience induced a persistent DNA hypermethylation in the medial prefrontal cortex. Intracortical inhibition of DNA methylation 1 month after training provoked forgetting, but not when the inhibitors were infused 1 day after conditioning (Miller et al., 2010).

Several studies reported changes associated with the process of forgetting. For instance, Tellez et al. (2012) found that forgetting is associated with modifications in some neural transporters in different brain regions including the hippocampus. It would be interesting to determine whether these neural transporters are required for active forgetting.

## Direct Evidence for Active Forgetting

Although compelling and indirect evidence strongly support the hypothesis that nervous system has the property of actively erasing stored information, some direct evidence for the existence of such a function or mechanism in the brain is needed. Main predictions to consider direct evidence for active inherent forgetting is to find that the specific inhibition of that mechanism will promote memory storage maintenance and the selective stimulation of it will provoke forgetting. Some molecular mechanisms that become strong candidates to be part of active intrinsic forgetting like the synaptic removal of GluA2 receptors or the activity of some phosphatases (Genoux et al., 2002; Migues et al., 2010, 2016; Sachser et al., 2016) partially accomplish the above-mentioned predictions. In addition, both predictions need additional requirements in order to consider them appropriately fulfilled. Confounds factors like facilitation or inhibition of memory formation have to be experimentally ruled out in order to establish the existence of an inherent forgetting process. In my opinion, to avoid some confounds it is preferable to study intrinsic mechanisms of forgetting in consolidated memories. A good example of such requirements is the findings of GluA2 endocytosis in non-consolidated short-term inhibitory avoidance memory (Dong et al., 2015). Only when information endorsing both predictions and requirements is available, one can be sure that a mechanism of time-dependent active forgetting is present. To the best of our knowledge these predictions and requirements were totally achieved by a few molecular mechanisms in *Drosophila* and by one process in the rat (see below).

In the following section I will review the scarce direct evidence regarding active inherent forgetting in brain circuits obtained so far. Some of the data mainly come from invertebrate (*Drosophila*) models of early unconsolidated memory, and the rest is provided by studies in consolidated memory in rodents. Therefore I will discuss first intrinsic mechanisms of active forgetting in *Drosophila* and then those observed in mice and rats.

### Active Forgetting in *Drosophila*

**Figure 1:**
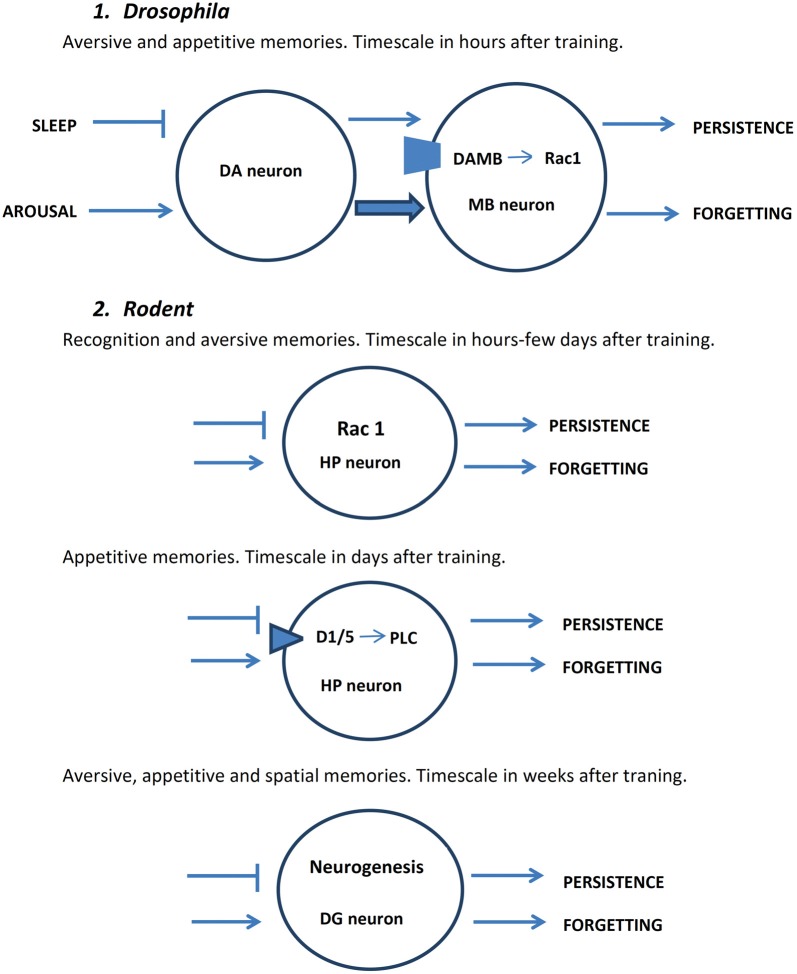
Schematic representation of major mechanisms of active forgetting in *drosophila* and rodents. DA, dopamine; DAMB, dopamine receptor in mushroom body (MB) neuron; D1/5, dopamine receptor subtype in mammals; DG, dentate gyrus; HP, hippocampus; PLC, phospholipase C. 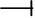 represents inhibition; → represents facilitation; arrows at the right part of neurons represent behavioral outcome.

Using a well-characterized olfactory aversive conditioning in *Drosophila*, the group of Zhong and colleagues (Shuai et al., 2010) showed that Rac1, a member of the Rho family of GTPases, is major player of active forgetting of early aversive memory. Rac is important for cytoskeleton dynamics and regulates synaptic spines through the modulation of actin polymerization (Heasman and Ridley, 2008). Inhibition of Rac1 induced a decrease in memory decay increasing its duration from a few hours to more than 1 day (see Figure 1). In contrast, stimulation of Rac activity induced forgetting of odor-shock association (Shuai et al., 2010). Therefore, Rac activity fulfills the main requisite to be considered a key step in active forgetting of early olfactory aversive memory in *Drosophila*. Hyperactivation of cofilin, a well-established downstream target of Rac1, enhanced early memory while inhibition of cofilin attenuated memory (Shuai et al., 2010).

Subsequently, the group of Ron Davis (Berry et al., 2012) reported that DA in the mushroom bodies of the fly has two functions: memory formation and forgetting. They studied 3 h memory retention of olfactory classical conditioning and found that a small subset of DA neurons, via posttraining activation of a DA receptor called dopamine receptor in mushroom body (DAMB) localized to intrinsic neurons of the mushroom bodies, are critical for active forgetting of early labile memory (see Figure 1). In addition, activation of these dopaminergic neurons induced forgetting of consolidated aversive as well as appetitive memories as tested at 6 h after training. DA via activation of another receptor dDA1 which is the *Drosophila* homolog of mammalian D1 receptor is required for aversive and appetitive memory acquisition (Kim et al., 2007). The authors proposed that after a new memory is formed both memory consolidation and active forgetting coexist, and that ongoing activity in a subset of dopaminergic neurons begins to erode what is being formed. Based on these considerations it is tempting to suggest that relevant or salient information to be stored enhances consolidation processes and/or attenuates active forgetting mechanisms.

Interestingly enough is that sleep after learning improves memory by blocking DA-based forgetting and arousal accelerates forgetting by increasing dopaminergic signaling due to sensory stimulation (Berry et al., 2015). It appears also that parallel systems exist that are involved in active forgetting: additional subset of DA cells and a couple of glutamatergic neurons were found to participate in olfactory aversive memory loss (Shuai et al., 2015). More recently it has been demonstrated that Scribble, a scaffolding protein that interacts with Rac1, Pak, and cofilin in mushroom body (MB) neurons, is required for forgetting of olfactory memories and is part of a signaling pathway triggered by DA acting on DAMB receptors (Cervantes-Sandoval et al., 2016). These findings help understand how ongoing DA activity at MB neurons may regulate actin dynamics and cytoskeleton rearrangements to induce active forgetting.

Another member of Rho GTPases family is Cdc42. Importantly, it has been recently demonstrated that this small G protein is involved in active forgetting of consolidated olfactory aversive memory (Zhang et al., 2016). Conditioning activates this protein and induces forgetting without altering its formation while the expression of a dominant negative mutant of Cdc42 increases persistence of memory storage (see active forgetting in rodents). This finding highlights the idea that each phase of memory processing olfactory aversive conditioning in *Drosophila* is under the control of different forgetting mechanisms (Table 1).

**Table 1:** Mechanisms of memory forgetting: behavioral effects of inhibition or facilitation of molecular events involved in active forgetting.

Animal target	Learning task	Memory stage	Inhibition	Facilitation
*Drosophila* Rac1	Olfactory aversive conditioning	Early	Persistence	Forgetting
*Drosophila* Dopamine neurons	Olfactory aversive or appetitive conditioning	Early (3 h)	Persistence of early memory (6 h)	Forgetting
*Drosophila* Dopamine receptor DAMB	Olfactory aversive conditioning	Early (3 h)	Persistence (Up to 24 h)	ND
*Drosophila* scribble	Olfactory aversive conditioning	Early	Persistence (up to 24 h)	ND
*Drosophila* Cdc42	Olfactory aversive	Intermediate	Persistence of Anesthesia-resistant-memory	Forgetting
Mouse Rac1	Object recognition	LTM (24 h)	Increased duration (up to 5 days)	Forgetting
Rat Dopamine receptor (D5?)	Conditioned place preference (appetitive)	LTM	Persistence of LTM (up to 14 days)	Forgetting
Mouse Adult neurogenesis	Aversive and appetitive tasks	LTM	Persistence of LTM (up to 6 weeks)	Forgetting

### Active Forgetting in Rodents

1. The role of Rac 1 in rodents is at least controversial. It is important to stress here that is difficult to compare the type and stages of memory processing in rodents with those in invertebrates (Davis, 2011). Only two recent works seem to study specifically whether this small G protein is involved in active forgetting. Liu et al. (2016) found that activation of Rac1 activity in hippocampal neurons accelerates forgetting of an object recognition task in mice: a memory that normally persists for more than 24 h but less than 72 h now with the expression of constitutive active Rac1 it is maintained less than 24 h. In contrast, inhibition of Rac1 activity prolongs the duration of object recognition memory up to 5 days (Figure 1). Unexpectedly, no changes in memory persistence were found in contextual fear conditioning and in trace fear conditioning (Liu et al., 2016). These findings suggest that Rac1-mediated forgetting is not a general mechanism of time-dependent memory loss.

Using massed and spaced contextual fear conditioning in rats Jiang et al. (2016) demonstrated that hippocampal inhibition of Rac1 with NSC27366 immediately after training facilitated memory retention in massed trained animals at 1 and 7 days, reaching similar freezing values to those obtained in spaced trained rats. The activation of Rac1 resulted in partial amnesia at the same time points. Together these findings suggest that Rac1 in the rat HP may be important for active forgetting of early fear memory. It would be interesting to determine whether inhibition of Rac1 affects immediate and short-term contextual fear memory.

Rac1 and its downstream effector cofilin also modulate 1-day cocaine-associated memory in conditioned place preference task. While activation of Rac1 in nucleus accumbens impaired memory, inhibition of Rac1 potentiated memory performance (Dietz et al., 2012). Unfortunately, this study did not include experiments to determine whether Rac1 is involved in active inherent forgetting to rule out the possibility that the abovementioned effects are merely due to modifications in the mechanisms of memory formation.

As it happened in *Drosophila*, it has been suggested that Cdc42 may participate in active forgetting. Cdc42 knockout mice exhibited normal acquisition and LTM retention performance in contextual fear conditioning and Morris water maze, but impaired remote memory recall (Kim et al., 2014). However, when a mechanism that induces forgetting is inhibited the main prediction is the increase in memory duration. Therefore, further experiments are needed to understand the role of Cdc42 in mammalian memory.

2. In the rat HP there are active molecular processes that fulfill the requirement to consider them as rapid inherent time-dependent forgetting mechanism. First, blocking hippocampal D1/D5 DA after learning induced a long-lasting cocaine-associated LTM extending the durability of the normally short-lasting LTM (single-trial conditioned place preference). This training is associated with delayed increase in DA levels in the dorsal HP (Kramar et al., 2014). In contrast, a D5 receptor agonist, but not a D1 receptor agonist, induced rapid forgetting of the normally long-lasting LTM (multiple-trial conditioning; Kramar et al., 2014). The time-dependent memory loss effect on this positive-valence memory by dopaminergic stimulation of the dorsal HP is opposite to those effects obtained in two different negative-valence memories (inhibitory avoidance and conditioned place aversion): D1 receptor agonist induced BDNF-dependent persistence of LTM (from a short-lived memory that lasts a couple of days to a long-lived memory lasting more 2 weeks), whereas hippocampal D1/D5 receptor inhibition induced memory loss (Figure 1; Rossato et al., 2009; Kramar et al., 2014). Therefore, in both species *Drosophila* and rats DA is crucial for active time-dependent forgetting. While in *Drosophila* DA through the receptor DAMB is crucial for active forgetting of early non-consolidated memories of negative- and positive-experiences (Davis and Zhong, 2017), in rats two different hippocampal DA receptors, adenylyl cyclase-coupled D1 R and D5 R (phospholipase C (PLC)-coupled D1 receptor), participate in active forgetting processes of negative and positive valence memories, respectively.
3. Finally, recent experimental evidence support the idea that adult neurogenesis in the HP bidirectionally regulates active natural forgetting (Figure 1). For instance, voluntary exercise during 6 weeks after fear conditioning induced an increase in neurogenesis and attenuated contextual fear conditioning and other HP-dependent learning tasks in mice such as inhibitory avoidance, water maze, Barnes maze and olfactory paired associate task (Akers et al., 2014; Epp et al., 2016). In contrast, inhibition of hippocampal neurogenesis decreased forgetting after a 6 week period following a water maze training (Epp et al., 2016). Together, these findings endorse the idea that adult neurogenesis in the HP play an important role in making room for new information (see Table 1).

### Active Mechanisms That Constrain Memory Formation

In addition to intrinsic mechanisms of forgetting some other brain mechanisms may constrain the formation and consolidation of memories. The result of their blockade induces facilitation of memory retention and/or an increase in the duration of a given memory. Their stimulation may down-regulate memory strength or durability. Therefore, they appear not to be constituents of active inherent forgetting processes, but may well help limit the stability of new memories. The following are the best characterized constraints:

1. Calcineurin and protein phosphatase 1 are inhibitory constraints that normally down-regulated the formation and maintenance of nonassociative as well as associative learning tasks (Mansuy et al., 1998; Genoux et al., 2002; Baumgärtel et al., 2008). The expression of a calcineurin inhibitor enhances short-term and long-term object location and object recognition memories (Malleret et al., 2001). Acquisition and memory storage of a spatial training in a Morris water maze is also enhanced. In a similar way, positive valence memory is facilitated (Gerdjikov and Beninger, 2005). The level of calcineurin in the amygdala at the moment of aversive memory modulates the strength of a memory (Baumgärtel et al., 2008). Calcineurin may limit several key signaling pathways including Ca^2+^-dependent, adenylyl cyclases-dependent and protein kinases-dependent processes acting on ion channels, glutamate receptors, and transcription factors (Winder and Sweatt, 2001; Oliveria et al., 2007). Inhibition of PP1 prolongs remote, but not recent spatial memory when induced after learning, suggesting that PPI may promote active forgetting (Genoux et al., 2002). However, no experiments were done to establish whether activation of PPI is sufficient to active forgetting. A similar situation is established also with calcineurin (Sachser et al., 2016).
2. Chen et al. (2003) used transgenic mice with a dominant-negative inhibitor against the transcription factors ATF4 and C/EBP and showed an increase in the duration of spatial memory switching a short-lived memory built up by using a weak training protocol to a long-lived spatial memory, which is consistent with the idea that these nuclear transcription factors are constraints of establishing remote memory. As previously shown, epigenetic modifications of histone deacetylase activity are constrains of consolidated LTM storage; inhibition of these enzymes in the dorsal HP caused weak objected recognition memory to persist (Federman et al., 2013).

### Processes Associated with Changes in the Rate of Forgetting

1. Aging is commonly associated with rapid forgetting. Aged and young rodents have similar retention scores in many learning tasks when testing is about 1 day after training, but when testing occurs several days after aged rats and mice have poor memory (see for references Countryman and Gold, 2007). Rate of forgetting is associated with the activation of transcription factors like CREB. This is consistent with results obtained in spatial memory of aged mice with a genetic inhibition of PP1 that exhibit an increase in CREB transcriptional activity (Genoux et al., 2002).
2. Infantile amnesia. Natural forgetting of episodic, HP-dependent memories occurring during the first 3–4 years of our life (Hayne, 2004). A similar phenomenon is also present in other mammals, like rodents (Campbell and Spear, 1972). Both HP-dependent and independent memories acquired during a critical time period in the rat are rapidly forgotten (see for references Travaglia et al., 2016; Alberini and Travaglia, 2017). One of the two main hypotheses to explain this infantile amnesia is that during the first 3 years the rate of neurogenesis in the dentate gyrus (DG) of the hippocampal formation is very high compared to the adult brain and given the role of neurogenesis in forgetting (Feng et al., 2001; Akers et al., 2014), infantile amnesia is probable due to memory loss.
3. Acute stressful experiences during a critical late consolidation period after training bidirectionally regulate durability of fear-motivated HP-dependent LTM.

Cold water stress promoted the duration of short-lasting fear LTM from 1 day to more than 7 days (Yang et al., 2013). Administration of corticosterone had similar effects and the inhibitor of corticosterone synthesis metyrapone blocked stress and corticosterone-induced persistence of LTM. In addition, exposure to a novel, but not familiar, open field which rapidly elevates corticosterone levels (Handa et al., 1994), induced a long-lasting inhibitory avoidance memory in rats trained with a weak protocol that normally gives short-lasting LTM (Tomaiuolo et al., 2015). This promoting effect of spatial novelty on memory persistence is time-dependent, does not alter memory consolidation and requires D1/D5 DA receptors activation and Arc expression.

On the other hand, when we trained rats with strong inhibitory avoidance protocol that leaves long-lived avoidance LTM lasting 2–4 weeks, exposure to a novel environment induced rapid forgetting (Katche et al., 2016). Therefore, environmental factors can modify the duration of consolidated fear memory in a time-restricted manner.

## Concluding Remarks

In the last 15 years emerges the idea that in addition to retroactive interference, retrieval-induced forgetting and passive decay of time (Anderson, 2003; Wixted, 2004), there are several processes in the nervous system of invertebrates and vertebrates that control what is stored and what is forgotten. These processes operate at different timescales and in different memory types. Some works immediately after acquisition at an early stage of memory formation and involves DA-releasing neurons, specific DA receptor subtypes, Rac1 activation, modifications in actin cytoskeleton at dendritic spines, and induces rapid active forgetting. Others regulate forgetting and persistence of seemingly consolidated memories modifying the activity of DA inputs to the HP and related brain regions activating parallel signaling cascades to induce active forgetting. Most of them may involve plastic changes in synaptic and extrasynaptic receptors including specific removal of GluA2 AMPA receptors. In addition, adult neurogenesis in the HP may induce forgetting at longer timescales. These are new and exciting examples of how brains are endowed with specific mechanisms to erase memory storage.

## Author Contributions

The author confirms being the sole contributor of this work and approved it for publication.

## Conflict of Interest Statement

The author declares that the research was conducted in the absence of any commercial or financial relationships that could be construed as a potential conflict of interest.
